# Forecasting type-specific seasonal influenza after 26 weeks in the United States using influenza activities in other countries

**DOI:** 10.1371/journal.pone.0220423

**Published:** 2019-11-25

**Authors:** Soo Beom Choi, Juhyeon Kim, Insung Ahn

**Affiliations:** 1 Department of Data-centric Problem Solving Research, Korea Institute of Science and Technology Information, Daejeon, Republic of Korea; 2 Center for Convergent Research of Emerging Virus Infection, Korea Research Institute of Chemical Technology, Daejeon, Republic of Korea; Newcastle University, UNITED KINGDOM

## Abstract

To identify countries that have seasonal patterns similar to the time series of influenza surveillance data in the United States and other countries, and to forecast the 2018–2019 seasonal influenza outbreak in the U.S., we collected the surveillance data of 164 countries using the FluNet database, search queries from Google Trends, and temperature from 2010 to 2018. Data for influenza-like illness (ILI) in the U.S. were collected from the Fluview database. We identified the time lag between two time-series which were weekly surveillances for ILI, total influenza (Total INF), influenza A (INF A), and influenza B (INF B) viruses between two countries using cross-correlation analysis. In order to forecast ILI, Total INF, INF A, and INF B of next season (after 26 weeks) in the U.S., we developed prediction models using linear regression, auto regressive integrated moving average, and an artificial neural network (ANN). As a result of cross-correlation analysis between the countries located in northern and southern hemisphere, the seasonal influenza patterns in Australia and Chile showed a high correlation with those of the U.S. 22 weeks and 28 weeks earlier, respectively. The *R*^2^ score of ANN models for ILI for validation set in 2015–2019 was 0.758 despite how hard it is to forecast 26 weeks ahead. Our prediction models forecast that the ILI for the U.S. in 2018–2019 may be later and less severe than those in 2017–2018, judging from the influenza activity for Australia and Chile in 2018. It allows to estimate peak timing, peak intensity, and type-specific influenza activities for next season at 40^th^ week. The correlation between seasonal influenza patterns in the U.S., Australia, and Chile could be used to forecast the next seasonal influenza pattern, which can help to determine influenza vaccine strategy approximately six months ahead in the U.S.

## Introduction

Seasonal influenza viruses are a significant public-health problem that causes a great many deaths worldwide every year [1]. The annual recurrence of seasonal epidemics is attributed to the continued evolution of seasonal influenza viruses, which enables them to escape the immunity that is induced by prior infections or vaccinations, and to the ability of those viruses to be transmitted efficiently human to human via respiratory droplets, direct contact, and fomites [1]. Currently, the human influenza A subtypes H1N1 and H3N2, as well as influenza B viruses, are the most commonly encountered variants worldwide [2]. Vaccination for seasonal influenza is the primary tool for reducing its morbidity and death rate [3].

Early prediction of the international spread of viruses during a potential pandemic can guide public-health actions globally [4]. Several previous studies have focused on predicting the incidence rate, peak time, or onset time of influenza-like illness (ILI) using data from online volunteer participants, ILI-related queries on Google, Wikipedia logs, or a combination of several data sources, including temperature and humidity [5–7]. However, the previous studies have focused on short-term forecasts for up to four weeks [5–7], and few studies have predicted seasonal influenza epidemics by using influenza information from neighboring regions [8,9].

Because influenza epidemics are acute, the long-term circulation of influenza viruses in the human population is driven by the global movement of viruses [1]. The extent to which viruses move internationally versus persisting locally during different epidemics has been of interest since at least the 1800s [1]. High-quality influenza surveillance systems are needed to enable countries to better understand influenza epidemiology, including disease incidence and severity, and help them implement appropriate prevention strategies [10].

The time horizon for which predictions are generally offered is in the order of 2 to 4 weeks, which is generally too short for action [11]. Because of the time-consuming nature of vaccine production, vaccine strategy needs to be prepared at least six months in advance of the upcoming flu season [12]. The optimal strategy for any country is to use the most recently available vaccine formulation with respect to local peak influenza timing, as long as the vaccine is available at least two months prior to the peak (early October for the Northern Hemisphere formulation) [13].

Previous studies forecasting the next seasonal influenza were based on the previous influenza patterns using time-series prediction models, such as the autoregressive integrated moving average (ARIMA) model or a simple humidity-forced susceptible–infectious–recovered–susceptible mathematical model [14,15]. Although the influenza season occurs annually, unique characteristics particular to each influenza season make forecasting difficult. Each year, the geographical location, rates of increase and decrease, duration, and size of each outbreak vary considerably [16]. Statistical models using historical data may accurately describe the typical pattern for a particular year, but they do not predict departures from the norm [16]. Therefore, it is important to find factors related to the next seasonal influenza pattern and to make a long-term prediction model.

To identify countries with seasonal patterns and influenza outbreaks that are similar to but precede those of the United States, we used FluNet surveillance data to investigate cross–correlation between the U.S. and other countries, in terms of time-series of the ILI, total influenza (Total INF), influenza A (INF A), and influenza B (INF B) viruses. Our hypothesis was that similar seasonal patterns of influenza outbreaks between two countries over the years are associated with influenza activity in the following year. Knowing about such an association may help clinicians to predict the pattern of influenza incidence in the next season. The prediction model allows to estimate peak timing, peak intensity, and type-specific influenza activities for next season at 40^th^ week.

## Methods

### Data collection

Data for ILI in the U.S. were collected from the Fluview database of the Centers for Disease Control and Prevention in the U.S. The agency’s website (https://gis.cdc.gov/grasp/fluview/fluportaldashboard.html) provides both new and historical data. The CDC’s ILI is freely distributed and available through ILInet [17]. From this website, we can obtain the CDC’s dataset on weighted ILI (%).

Influenza surveillance data were collected from the FluNet database of the WHO Global Influenza Surveillance Network (URL: http://apps.who.int/flumart/Default?ReportNo=12) [3, 18]. These data are uploaded to the FluNet database every week by the countries in the network [3]. The FluNet database contains the following variables, reported by 164 countries: influenza transmission zone, number of specimens, number of INF A and INF B detected by subtype, and number of influenza-positive viruses. We collected the surveillance data of the 164 countries from the 40^th^ week in 2010 to the 40^th^ week in 2018. We set the starting point for our research data in 2010, because influenza season during 2009 was an atypical season, with the introduction of a novel pandemic strain (INF A H1N1 pdm09) [19]. Missing data were replaced by a zero because some countries conducted surveillance only during weeks with high influenza activity. Total INF, INF A, and INF B were defined as the number of influenza subtypes detected among processed specimens.

Google Trends (GT) (https://trends.google.com/trends/) is the principal tool used to study the trends and patterns of Google search engine queries [20]. Google Trends provides search activity for each country and specific time periods. We included the search topics, “influenza”, “influenza A virus”, and “influenza B virus” in the U.S., Australia, and Chile from October 2010 to October 2018. The search queries data from Google Trends were linearly interpolated from monthly data to weekly data points.

**Fig 1:**
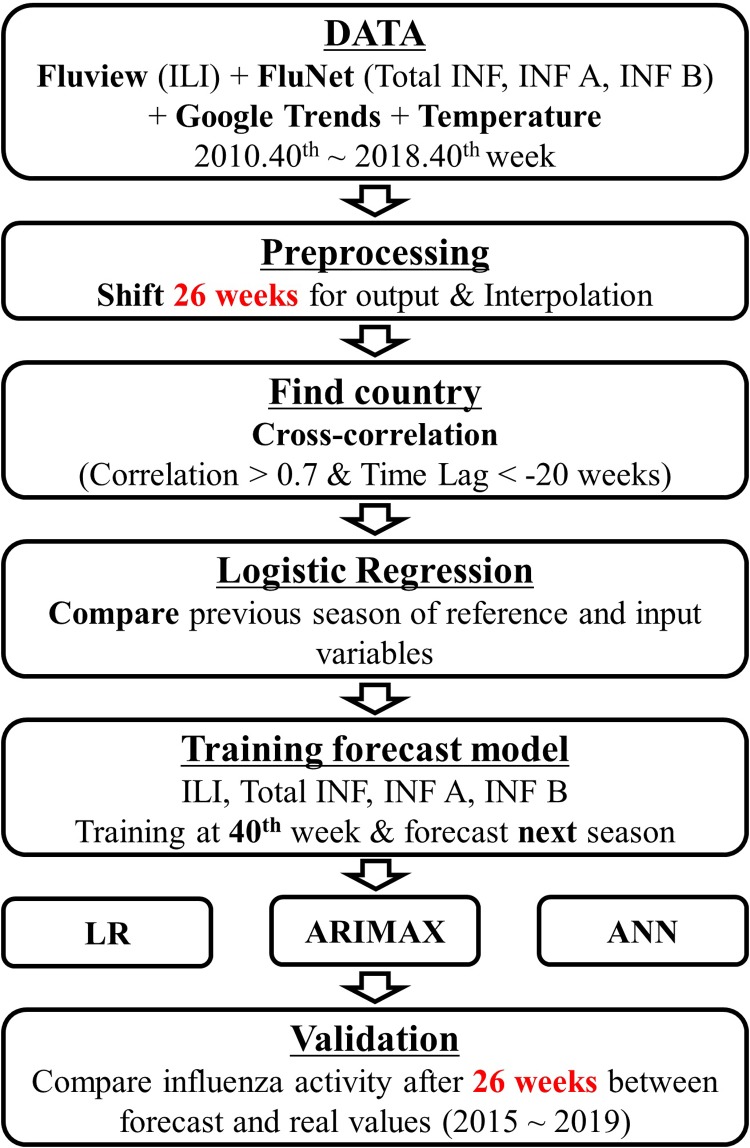
The flow chart for this study.

The daily temperature data in the U.S. was obtained from the National Oceanic and Atmospheric Administration (ftp://ftp.ncdc.noaa.gov/pub/data/ghcn/daily/by_year/). We included weekly temperature data and the average values for all stations in the U.S. during a week. The overall flow chart for this study is presented in Fig 1.

### Statistical analysis

**Fig 2:**
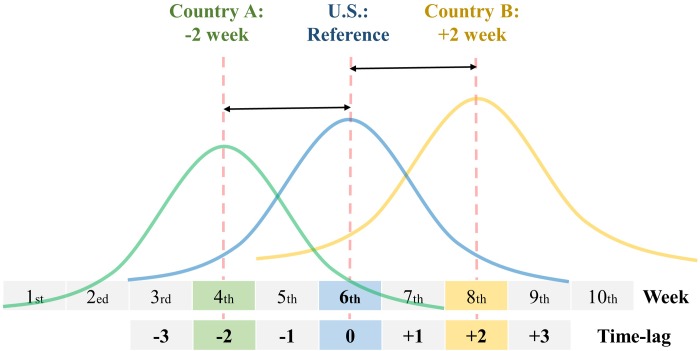
An example of cross-correlation analysis.

In two time periods, cross-correlations were analyzed using Pearson’s correlation, with a time lag range of ± 30 weeks, with Bonferroni’s correction. Cross-correlation allows the time lag between two time series to be identified [21]. If the blue waveform of the reference country correlates with the green waveform of country A with a time lag of -2 weeks, the peak or onset of the reference country can be identified as occurring 2 weeks later than that of country A (Fig 2). Time-series for ILI, Total INF, INF A, and INF B in the U.S. as reference were analyzed with the Total INF, Total INF, INF A, and INF B, respectively, in the comparison country using cross-correlation. For example, ILI in the U.S. was compared to Total INF in each comparison country. Moreover, cross-correlation analysis was also performed on the GT and temperature data. We selected countries with a time lag between -20 and -30 weeks and a correlation coefficient of 0.7 or more in Table 1.

**Table 1:** Maximum correlation coefficient and time lag with time series of influenza surveillance data in the United States and input variables.

ILI			Total INF			INF A			INF B		
Country	Corr	TL	Country	Corr	TL	Country	Corr	TL	Country	Corr	TL
Canada	0.891	1	Australia*	0.892	-22	Australia*	0.896	-22	Norway	0.884	0
Australia	0.861*	-22	Canada	0.885	1	Canada	0.830	0	Sweden	0.864	1
Germany	0.790	4	U.K	0.826	0	Chile*	0.803	-28	Croatia	0.856	-2
Chile	0.786*	-28	Norway	0.804	2	U.K	0.747	0	U.K	0.855	-2
Norway	0.778	2	Denmark	0.796	4	Kuwait	0.701	-9	Canada	0.851	0
Sweden	0.762	3	Chile*	0.778	-29	Myanmar	0.677	-23	Denmark	0.848	1
Iceland	0.756	3	Spain	0.775	-2	Bangladesh	0.657	-30	Switzerland	0.841	-4
Japan	0.741	-1	Iceland	0.770	2	Luxembourg	0.655	4	Ireland	0.840	-3
Croatia	0.731	1	Sweden	0.767	3	Laos	0.651	-19	Italy	0.830	-4
U.K	0.718	1	Malta	0.766	-2	Oman	0.647	-11	Australia*	0.792	-25
GT_INF_U.S.	0.880	-1	GT_INF_U.S.	0.902	-1	GT_INF_U.S.	0.891	0	GT_INF_U.S.	0.834	-4
GT_INF A_U.S.	0.861	-2	GT_INF A_U.S.	0.934	-1	GT_INF A_U.S.	0.929	-1	GT_INF A_U.S.	0.864	-4
GT_INF B_U.S.	0.797	1	GT_INF B_U.S.	0.907	1	GT_INF B_U.S.	0.874	2	GT_INF B_U.S.	0.947	-2
GT_INF_Australia	0.596	-27	GT_INF_Australia	0.598	-26	GT_INF_Australia	0.568	-25	GT_INF_Australia	0.643	-29
GT_INF A_Australia	0.808*	-24	GT_INF A_Australia*	0.917	-24	GT_INF A_Australia*	0.899	-23	GT_INF A_Australia*	0.911	-27
GT_INF B_Australia	0.578	-23	GT_INF B_Australia	0.653	-24	GT_INF B_Australia	0.600	-23	GT_INF B_Australia*	0.710	-26
GT_INF_Chile	0.572	11	GT_INF_Chile	0.650	8	GT_INF_Chile	0.610	9	GT_INF_Chile	0.700	4
GT_INF A_Chile	0.800*	-30	GT_INF A_Chile*	0.767	-30	GT_INF A_Chile*	0.800	-30	GT_INF A_Chile	0.507	-30
GT_INF B_Chile	0.593	-23	GT_INF B_Chile	0.603	-24	GT_INF B_Chile	0.567	-23	GT_INF B_Chile	0.579	-26
Temp_U.S.	0.764*	-27	Temp_U.S.	0.568	-28	Temp_U.S.	0.560	-27	Temp_U.S.	0.510	-30

Corr, maximum correlation coefficient; GT, Google Trends; INF, Influenza; ILI, Influenza-like illness; Temp, temperature; TL, Time lag; U.K., United Kingdom of Great Britain and Northern Ireland; U.S., United States.

GT_INF_U.S. is GT with the keyword “influenza” in the U.S.

* Countries with a correlation coefficient of 0.7 or more and time lag between -20 and -30 weeks from 2010 to 2018.

Linear regression analysis (LR) was used to evaluate the relationship between influenza surveillances in the U.S. after 26 weeks and those in selected countries, and GT and temperature from the 40^th^ week in 2010 to the 40^th^ week in 2018. LR 1 used influenza data from the U.S. after 26 weeks as dependent variable and previous seasonal data from the U.S. as independent variable. LR 2 used influenza activities from selected countries, and LR 3 used GT with the keyword “influenza A virus” in selected countries. LR 4 used temperature in the U.S. The input variables in LR 5 were those of LR models with the adjusted *R*^2^ values higher than the LR 1, and these input variables were used to forecast seasonal influenza after 26 weeks in the U.S.

All statistical analyses were performed using Python 3.6.2 (Python Software Foundation), and *p* < 0.05 was considered statistically significant.

### Prediction model

**Fig 3:**
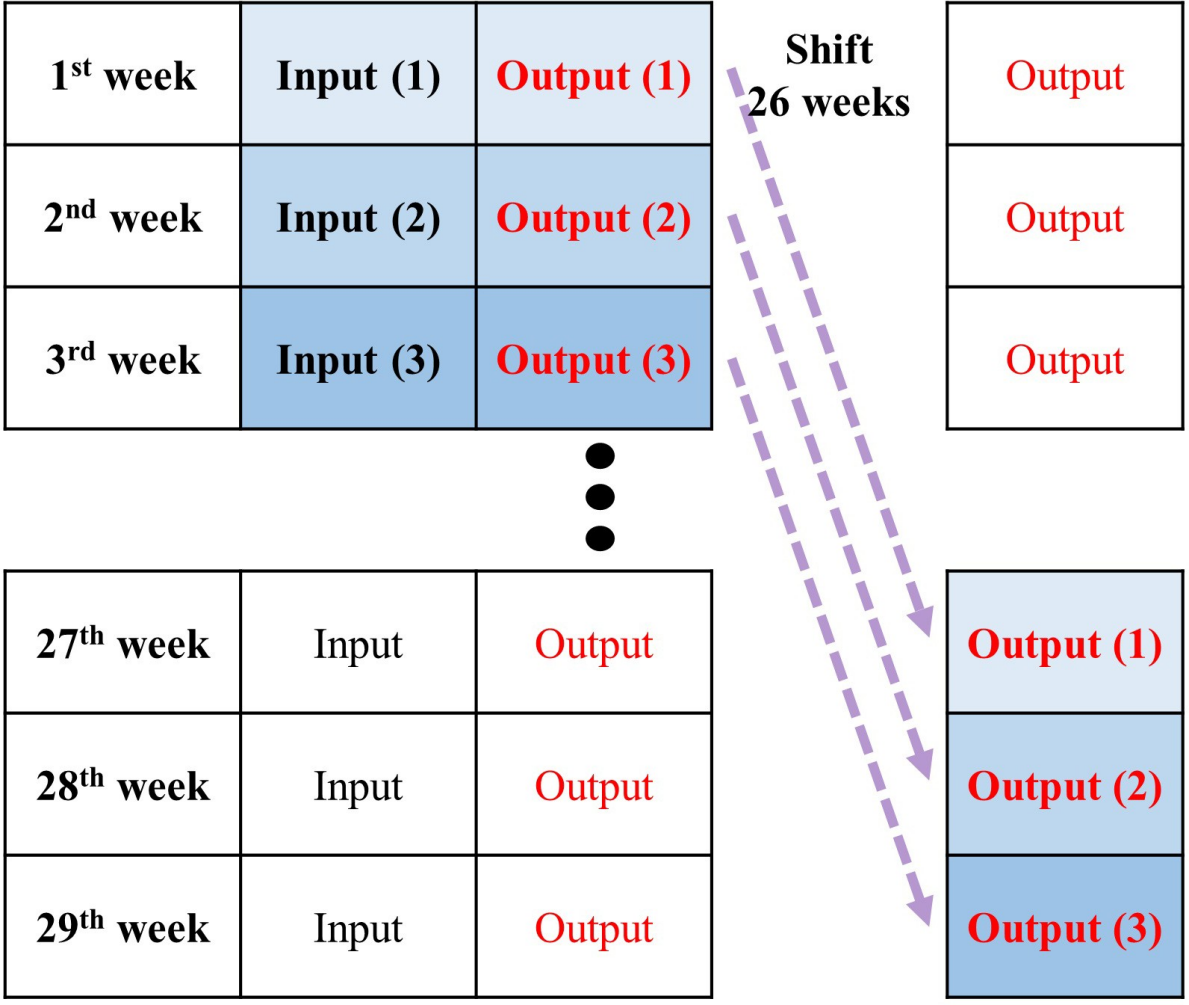
The explanation for forecasting seasonal influenza after 26 weeks.

**Fig 4:**
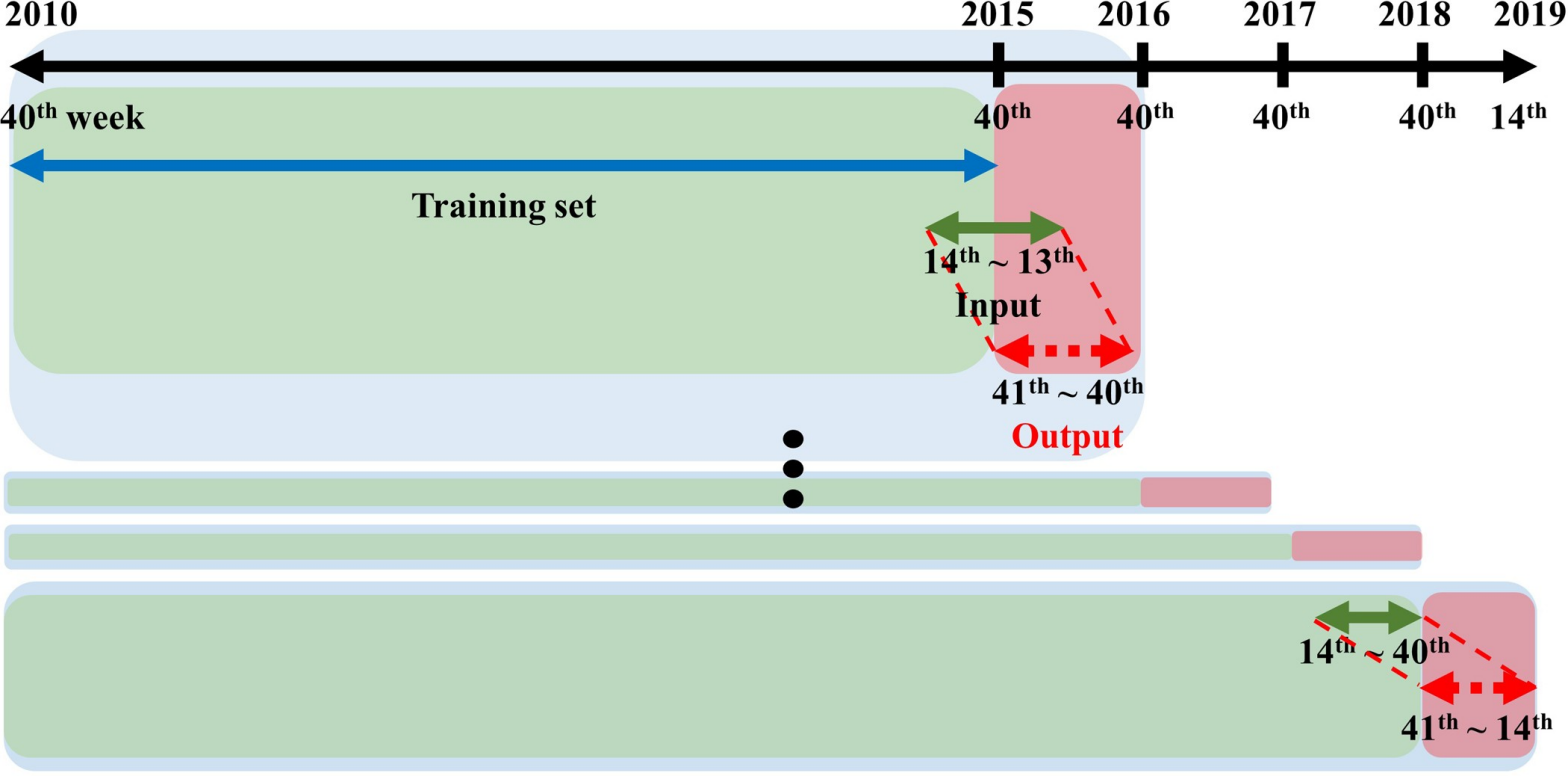
The explanation for the training set at the 40^th^ week and output for forecasting seasonal influenza after 26 weeks.

Forecasting seasonal influenza after 26 weeks was defined as forecasting influenza pattern after six months (26 weeks) with data available only at the current point (Fig 3). Four seasons for 2015–2019 were selected to validate the prediction models for seasonal influenza patterns. This is done because the training set accounted for 50% of eight seasons in the whole dataset for 2010–2018. For example, the prediction model for the seasonal influenza pattern during 2015–2016 used the influenza surveillance data from the 40^th^ week in 2010 to the 40^th^ week in 2015 as the training set (Fig 4).

The prediction model is a single-output model for seasonal influenza patterns after 26 weeks using the four historical values that indicate a month (4 weeks): we predict *Y*_t+26_ based on *X*_*t*_, *X*_*t*–1_, *X*_*t*–2_, and *X*_*t*–3_: *Y*, *X*, and *t* are the predicted value, input variable, and week, respectively [22].

In order to forecast ILI, Total INF, INF A, and INF B of next season (after 26 weeks) in the U.S., we developed prediction models using an artificial neural network (ANN), LR, and ARIMA including exogenous variables (ARIMAX). ANN is an artificial intelligence technology inspired by the architecture of biological neurons, such as that in the human brain [23]. The input layer receives an input signal, which moves to the next layer as a modified version of the input signal. It passes through several layers composed of multiple transformations, and last passes through the output layer as an output signal [24,25]. We implemented ANN using the python library Keras (version 2.2.0) with TensorFlow (version 1.8.0) backend. The scikit–learn library (https://scikit-learn.org/stable/) was used for data management and preprocessing. In this study, we used a three-layer ANN network with a 10% dropout rate for the first layer for the overfitting problem. The models were optimized using the Adam optimizer with a loss function of mean squared error. Neuron activation functions were rectified linear units for the second layer. We selected 100 epochs and one batch size for the ANN model.

Prediction models of LR were calculated to forecast influenza surveillance after 26 weeks in the U.S. per each year. The influenza time series is characterized by an autocorrelation, so we also employed the ARIMAX. The autocorrelation function and partial autocorrelation function is used to determine the autoregressive (AR) and moving average (MR) order. An ARIMA model is notated as ARIMA (*p*, *d*, *q*), where *p* indicates the AR order, *d* the differencing order and *q* the MA order [26]. For a complete description of the ARIMA analysis, see [27]. Prediction models of LR and ARIMAX were used the same input variables for ANN in S1 and S2 Tables, respectively.

### Validation for the prediction model

The coefficient of determination, *R*^2^, which corresponds to the percentage of the variance of the observed time-series that is explained by the model, was calculated. Root-mean-square error (RMSE) was calculated using real values and predicted values for influenza activities in the validation set from the 41^th^ week in 2015 to 14^th^ week in 2019. The onset of influenza weeks for ILI is defined as the weighted percentage that exceeds the national baseline [28]. The peak amplitude and the peak timing are defined as the maximum value and that week in seasonal influenza week.

## Results

### Cross–correlation analysis

Table 1 shows the maximum correlation coefficient and time lag between the seasonal influenza outbreaks in the U.S. and the input variables. The correlation coefficients were calculated by cross-correlation between ILI in the U.S. and the number of positive influenza viruses worldwide. In Table 1, Australia had the highest correlation coefficient of 0.896 (p = 1.24 ⅹ 10^−140^) with a -22 week time lag for the INF A. The -22 week time lag meant that the country’s seasonal influenza outbreak 22 weeks earlier was highly correlated with the seasonal influenza outbreak in the U.S.

In the analysis of the ILI and Total INF, the correlation coefficients of Australia were 0.861 with a -22 week lag and 0.892 with a -22 week lag, respectively. Chile had the third highest correlation coefficient of 0.803 (p = 6.82 ⅹ 10^−89^) with a -28 week time lag for the INF A. However, the correlation coefficients of Australia for the INF B were 0.792 with a -25 week lag, which is much lower than that for the INF A. Moreover, the correlations for the INF B in such European countries as Norway, Sweden, Croatia, and the UK were higher than those of Australia in the Southern Hemisphere. We selected Australia and Chile to forecast ILI, Total INF and INF A after 26 weeks in the U.S., and Australia was selected for INF B.

Although the correlation coefficients for GT with the keywords “influenza”, “influenza A virus”, and “influenza B virus in the U.S. showed high values, time lags for those ranged from -4 to 2 weeks, which were not eligible variables to forecast influenza after 26 weeks. However, GT with the keyword “influenza A virus” in Australia and Chile showed high correlation coefficients with -23 and -30 week time lags, respectively. The correlation coefficient for the ILI of temperature in the U.S. was more than 0.7 with a -27 week time lag.

**Fig 5:**
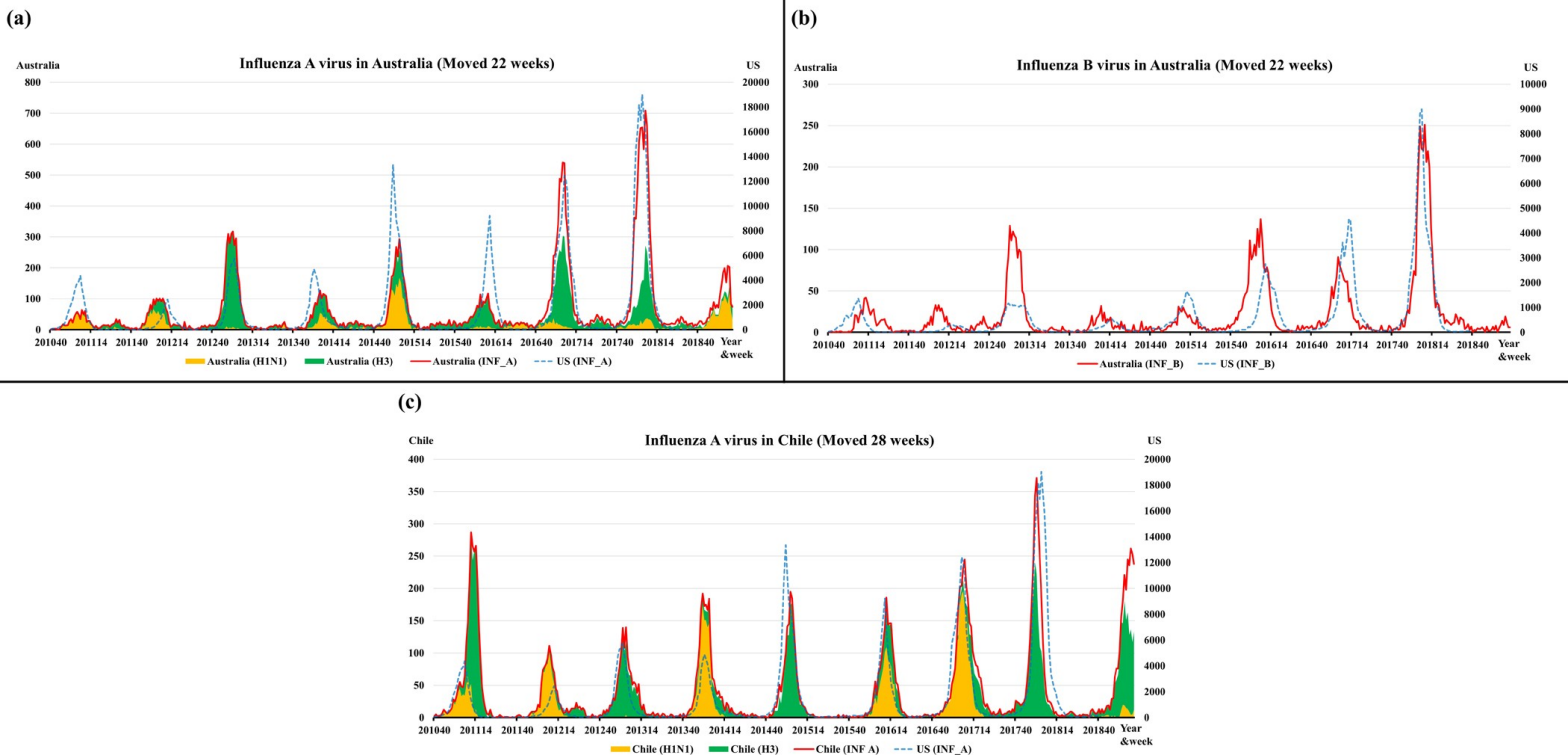
The surveillance data for influenza A (a) and B (b) viruses in the U.S. and Australia; the values for Australia were shifted forward 22 weeks in 2010–2018. The surveillance data for influenza A (c) viruses in the U.S. and Chile; the values for Australia were shifted forward 28 weeks. The blank part of the graph, the gap between INF A and the sum of the H1N1 and H3 viruses, is the number of influenza viruses (not subtyped).

Fig 5A and 5B, showing the INF A and INF B in the U.S. and Australia, shows that the values for Australia were shifted forward 22 weeks from the 40^th^ week in 2010 to the 40^th^ week in 2018. Interestingly, the waveforms for the U.S. and Australia are similar in peak timing and amplitude. Fig 5C, showing the INF A in the U.S. and Chile, shows that the values for Chile was shifted forward 28 weeks, the waveforms also similar. Therefore, influenza surveillance data in Australia and Chile could be valuable for predicting an influenza outbreak after 26 weeks in the U.S.

### Linear regression analysis

Table 2 shows results of LR for influenza after 26 weeks (ILI, Total INF, INF A, and INF B) in the U.S. using influenza activities for the previous season in the U.S, influenza activities in Australia and Chile, GT with the keyword “influenza A virus” in Australia and Chile, and temperature in the U.S. from 2010 to 2018. The results of LR 2 demonstrated that the adjusted *R*^2^ values for influenza surveillance at present in the selected countries were higher than those of the previous season in the U.S. in LR 1 for ILI, Total INF, INF A, and INF B. In LR 3, the adjusted *R*^2^ values for GT with the keyword “influenza A virus” in Australia and Chile were also higher than those of LR1 for ILI, Total INF, INF A, and INF B. The adjusted *R*^2^ values for temperature in the U.S. were only higher than that of LR1 for ILI. LR 5 shows the results for the final selection of input variables to forecast influenza activities after 26 weeks in the U.S.

**Table 2:** Linear regression analysis for influenza activities of previous season in the U.S. and input variables from the 40^th^ week in 2010 to the 40^th^ week in 2018.

ILI for the U.S. after 26 week					
	LR 1 Beta [P-value]	LR 2 Beta [P-value]	LR 3 Beta [P-value]	LR 4 Beta [P-value]	LR 5 Beta [P-value]
ILI—U.S. (before 26 week)	0.857 [<0.001]	-	-	-	-
Total INF—Australia (present)	-	0.005 [<0.001]	-	-	0.001 [0.485]
Total INF—Chile (present)	-	0.009 [<0.001]	-	-	0.007 [<0.001]
GT INF A—Australia (present)	-	-	0.059 [<0.001]	-	0.043 [<0.001]
GT INF A—Chile (present)	-	-	0.023 [<0.001]	-	-0.009 [<0.001]
Temp—U.S. (present)	-	-	-	0.111 [<0.001]	0.054 [<0.001]
Adj. R-squared	0.537	0.761	0.720	0.557	0.865
Total INF for the U.S. after 26 week					
ILI—U.S. (before 26 week)	1.076 [<0.001]	-	-	-	-
Total INF—Australia (present)	-	18.4 [<0.001]	-	-	-2.610 [0.012]
Total INF—Chile (present)	-	23.6 [<0.001]	-	-	26.8 [<0.001]
GT INF A—Australia (present)	-	-	246.7 [<0.001]	-	250.0 [<0.001]
GT INF A—Chile (present)	-	-	49.8 [<0.001]	-	-24.9 [0.001]
Temp—U.S. (present)	-	-	-	277.9 [<0.001]	-
Adj. R-squared	0.475	0.729	0.839	0.286	0.887
INF A for the U.S. after 26 week					
ILI—U.S. (before 26 week)	0.900 [<0.001]	-	-	-	-
Total INF—Australia (present)	-	16.3 [<0.001]	-	-	-3.415 [0.006]
Total INF—Chile (present)	-	25.3 [<0.001]	-	-	24.8 [<0.001]
GT INF A—Australia (present)	-	-	169.6 [<0.001]	-	182.7 [<0.001]
GT INF A—Chile (present)	-	-	56.2 [<0.001]	-	-7.491 [0.261]
Temp—U.S. (present)	-	-	-	219.9 [<0.001]	-
Adj. R-squared	0.387	0.690	0.769	0.300	0.832
INF B for the U.S. after 26 week					
ILI—U.S. (before 26 week)	1.344 [<0.001]	-	-	-	-
Total INF—Australia (present)	-	24.8 [<0.001]	-	-	9.636 [<0.001]
GT INF A—Australia (present)	-	-	74.9 [<0.001]	-	54.4 [<0.001]
Temp—U.S. (present)	-	-	-	58.0 [<0.001]	-
Adj. R-squared	0.552	0.627	0.749	0.143	0.787

Beta, Beta coefficient; CI, Confidence Interval; GT, Google Trends; INF, Influenza; ILI, Influenza-like illness; LR, Linear regression; Temp, temperature; U.S., United States of America.

GT INF A—Australia is GT with the keyword of “influenza A virus” in Australia.

### Prediction models

Table 3 shows the performance of the prediction models for seasonal influenza outbreaks in the U.S. after 26 weeks using previous season data, LR, ARIMAX, and ANN. The *R*^2^ scores of LR, ARIMAX, and ANN for ILI, Total INF, INF A, and INF B showed better performance than those of the previous season. The *R*^2^ score for the prediction model of ANN for ILI was 0.758. The *R*^2^ score of ARIMAX for Total INF was 0.806, which was the highest.

**Table 3:** Performance of prediction models for seasonal influenza outbreaks after 26 weeks in the United States from 2015 to 2019.

		R^2^	RMSE (forecast values)	RMSE (Peak timing)	RMSE (Peak amplitude)
Influenza-like illness	Previous season	0.487	1.1	5.5	2.2
	LR	0.720	0.8	2.5	1.5
	ARIMAX	0.714	0.8	2.9	1.6
	ANN	0.758*	0.7*	1.8*	0.8*
Total Influenza viruses	Previous season	0.346	4802.8	5.8	6657.8
	LR	0.792	2707.6	1.7	5574.5*
	ARIMAX	0.806*	2618.2*	1.7	6726.4
	ANN	0.738	3039.6	1.7*	7544.3
Influenza A virus	Previous season	0.289	4190.9	5.8	4901.0
	LR	0.777	2347.8	1.9	4694.7
	ARIMAX	0.792	2265.2	1.6	5355.9
	ANN	0.798*	2231.9*	1.2*	4545.3*
Influenza B virus	Previous season	-0.238	1880.8	3.7	5142.3
	LR	0.427*	1279.4*	3.0	1453.6*
	ARIMAX	0.352	1360.4	2.7	3163.0
	ANN	0.403	1306.3	2.4*	1512.0

R^2^, Coefficient of determination; RMSE, Root-mean-square error; LR, Linear regression; ARIMAX, Auto Regressive Integrated Moving Average; ANN, artificial neural network.

* Best value among previous season, LR, ARIMAX, and ANN.

Units for the RMSE (forecast values) are percentage of visits for influenza-like illness and number of total influenza, influenza A, and influenza B viruses.

Units for the RMSE (Peak timing) are week.

Units for the RMSE (peak amplitude) are percentage of visits for influenza-like illness and number of total influenza, influenza A, and influenza B viruses.

**Fig 6:**
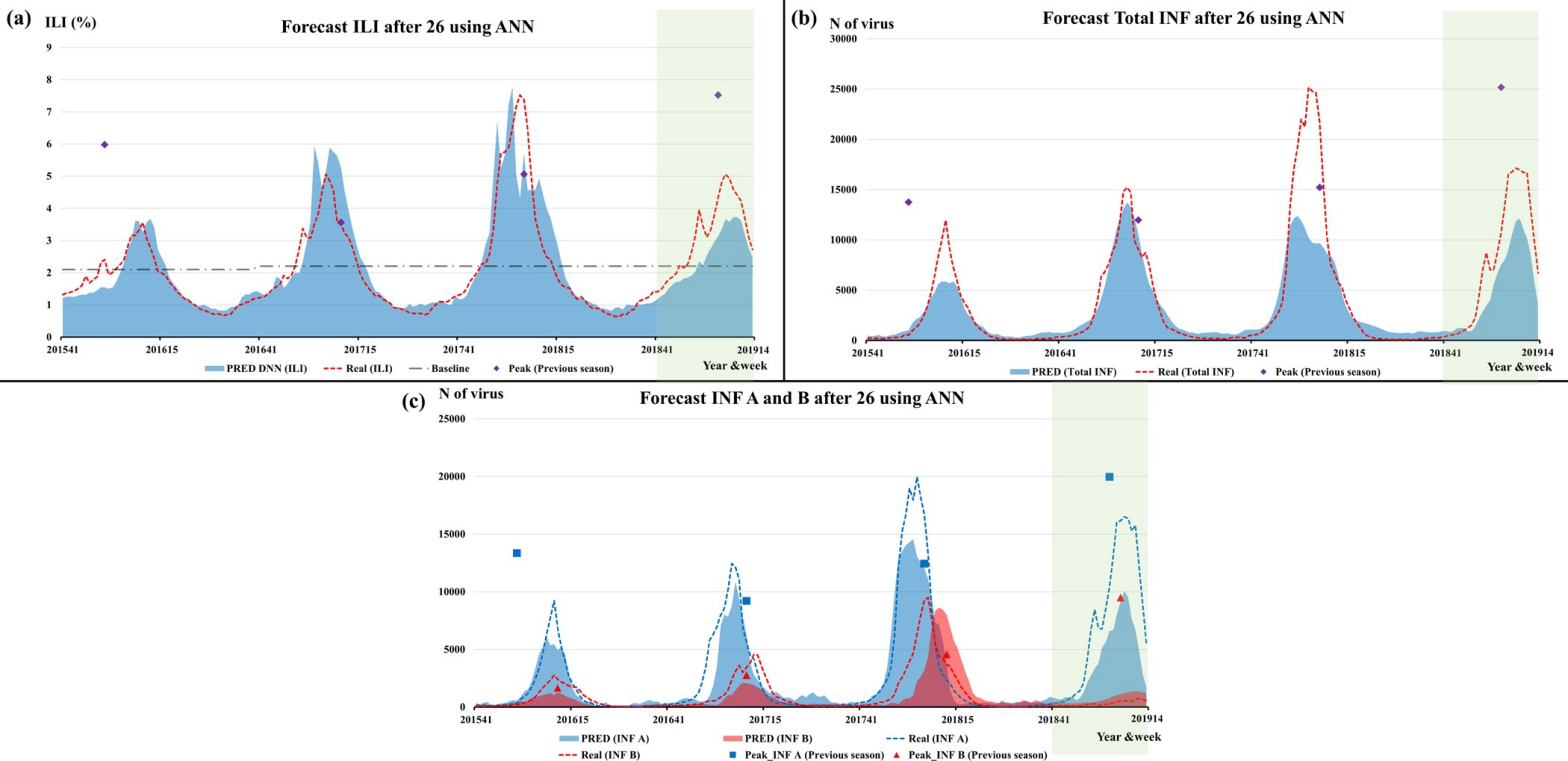
The prediction of ANN for ILI (a) and Total influenza (b), influenza A and influenza B viruses (c) after 26 weeks from 2015 to 2019 in the U.S.

**Fig 7:**
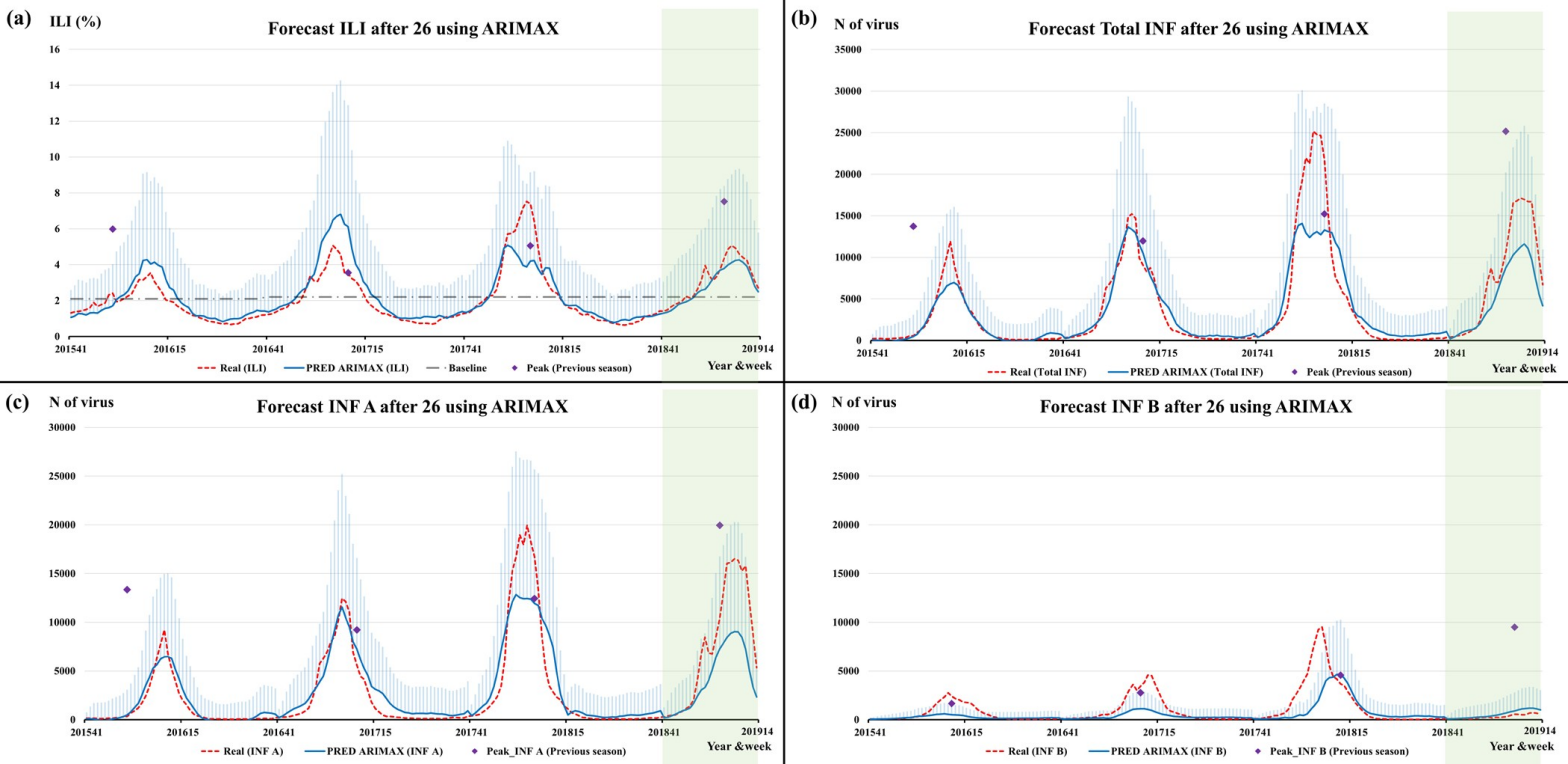
The prediction and 95% confidence interval of Auto Regressive Integrated Moving Average for ILI (a) and Total influenza (b), influenza A (c) and influenza B viruses (d) from 2015 to 2019 in the U.S.

Fig 6 shows the prediction of ANN for ILI, Total INF, INF A, and INF B from the 41^st^ week in 2015 to the 14^th^ week in 2019 in the U.S. using temperature in the U.S., surveillance data, and GT in Australia and Chile. Moreover, the forecast prediction by ARIMAX for type-specific influenza activities in the U.S. (blue line) and the 95% confidence interval (light blue bar) indicate the forecast uncertainty, as shown in Fig 7. The pattern of peak timing and amplitude of influenza activity for the 2015–2016 season was different from the 2012–2015 season, but our models forecasted later and less severe influenza activity. Moreover, the predicted INF B activity for the 2018–2019 season was a better match than the increasing pattern for the 2013–2018 seasons.

## Discussion

The aim of this study was to identify countries with seasonal patterns and influenza outbreaks that were similar to but preceded those of the United States. We used influenza activities in Australia and Chile, GT with the keyword “influenza A virus” in Australia and Chile, and temperature in the U.S. to forecast for seasonal influenza after 26 weeks in the U.S. The seasonal influenza patterns in Australia before 22 weeks and Chile before 28 weeks showed a high correlation with those of the U.S. In Table 2, influenza surveillance and GT in Australia and Chile at present were more useful than previous seasonal influenza in the U.S. for forecasting next seasonal influenza in the U.S. ANN models showed better performance for forecasting influenza activities after 26 weeks in the U.S than previous season data. The LR and ARIMAX models also showed high performance which can be interpretable. Our prediction models forecast that the ILI for the U.S. in 2018–2019 may be later and less severe than that in 2017–2018.

The seasonal influenza patterns in the U.S. were highly correlated with those in Canada, Australia, Chile, and the United Kingdom. The correlation coefficients for these countries were higher than those for their neighboring countries or for other countries in the Northern Hemisphere, for example, Mexico (0.412 for Total INF), Russia (0.329), Cuba (0.121), Spain (0.775), and Japan (0.600), which are not shown in Table 1. Moreover, the period of the primary influenza peak in the Southern Hemisphere countries was July and August [13], so the absolute values of the time lag for the U.S. in the Southern Hemisphere countries were more than 20 weeks in Table 1. However, the correlation coefficients for Argentina, Australia, Chile, and Uruguay with similar latitudes were different, at 0.516, 0.892, 0.778, and 0.303 for Total INF in the U.S., respectively, which are not shown in Table 1. For these reasons, the correlations for seasonal influenza between countries could be caused by characteristics of influenza, Economic Status, Educational Status, or Access to Health Care as well as by Season environmental factors in the Northern Hemisphere [3, 29]. However, this study did not prove the causality of correlations for seasonal influenza between countries. Although we do not know the reasons, the patterns for seasonal influenza in the U.S., Australia, and Chile were similar, and influenza surveillance in Australia and Chile can be used to predict seasonal influenza outbreaks after 26 week in the U.S.

A study of Bedford *et al*. demonstrated that the less-frequent global movement of INF A/H1N1 and B viruses coincided with slower rates of antigenic evolution, lower ages of infection, and smaller, less-frequent epidemics than for A/H3N2 viruses [30]. Their study analyzed the correlation of peak timing for seasonal influenza between countries, and gave a time-series graph for influenza surveillance from 2000 to 2012 in the U.S. and Australia [30]. The time-series of virological characterizations for A/H3N2 in the U.S. and Australia were consistent with the time-series graph in our Fig 5A. Bedford *et al*. suggested that differences in ages of infection could explain patterns of global circulation across a variety of human viruses [30].

Viboud *et al*. analyzed correlations for influenza epidemics from 1972 to 1997 in the U.S., France, and Australia [31]. In their study, France and the U.S. had a high correlation for influenza epidemics, but there was no significant correlation between the U.S. and Australia. In the scenario in which the influenza season in Australia was systematically six months in advance of that in the Northern Hemisphere, the median time lag between the peaks in Australia and in the United States was 27 weeks (range 14–39) [31]. Our study analyzed correlations for INF A and INF B, but the study of Viboud *et al*. did not [31].

The previous studies for prediction models for seasonal influenza have focused on social networking service data, search engine query data, and environmental factors [32–34]. These predictors are correlated with present influenza cases with a relatively short-term gap, of about one to four weeks. However, the influenza surveillance data in Australia had a time gap of 22 weeks from those in the U.S., which can help to establish a data-driven influenza vaccine strategy about six months ahead.

Kandula et al. analyzed whether forecasts targeted to predict influenza by type and subtype during 2003–2015 in the U.S. were more or less accurate than forecasts targeted to predict Total INF using four compartmental models [35]. They found that forecasts separated by type/subtype generally produced more accurate predictions and, when summed, produced more accurate predictions of Total INF [35]. Our prediction models for type-specific influenza is valuable as well as those for ILI, which could provide an important, richer picture of influenza activity.

To our best knowledge, this is the first study to use influenza surveillance in Australia and Chile for predicting influenza cases after 26 weeks in the U.S. However, our study has several limitations. For the ILI study, we could not use ILI data in other countries, instead had to use data for Total INF, because collecting ILI data separately for the 164 countries was difficult. There were not enough data on other potential covariates to show relationships between influenza outbreaks in Australia, Chile, and the U.S., such as the standard of the medical facilities, economic level, and medical records of the influenza virus. Furthermore, we included only data from laboratory-confirmed cases, which may underestimate the true incidence of influenza in the population [36]. Further research to explain underlying mechanisms for the relationship of influenza activities between these countries is warranted.

## Conclusions

Our study forecasts the 2018–2019 seasonal influenza after 26 weeks in the U.S. using the 2018 seasonal influenza in Australia and Chile. The correlation between the seasonal influenza patterns in the U.S., Australia, and Chile could be used to forecast the next seasonal influenza pattern, which can help to determine influenza vaccine strategy approximately six months ahead in the U.S. Our prediction model allows to estimate peak timing, peak intensity, and type-specific influenza activities for next season at 40^th^ week.

## Supporting information

S1 Table Linear regression models for influenza surveillance after 26 weeks in the U.S. (DOCX)

S2 Table Auto regressive integrated moving average including exogenous variables for influenza surveillance after 26 weeks in the U.S. (DOCX)
