# Supplementary material for: Forecasting type-specific seasonal influenza after 26 weeks in the United States using influenza activities in other countries
Source: PLoS One. 2019 Nov 25;14(11):e0220423. doi: 10.1371/journal.pone.0220423 (PMC6876883; doi:10.1371/journal.pone.0220423)
Supplement: S1 Table — (DOCX) [file pone.0220423.s001.docx]

**Supplementary Table 1.** Linear regression models for influenza surveillance after 26 weeks in the U.S.

| ILI for the U.S. after 26 week – Output Variable | | | | | | | | |
| --- | --- | --- | --- | --- | --- | --- | --- | --- |
|  | Model for 2016 using 2010-2015 | | Model for 2017 using 2010-2016 | | Model for 2018 using 2010-2017 | | Model for 2019 using 2010-2018 | |
| Adj.R-squared | 0.910 | | 0.891 | | 0.872 | | 0.898 | |
| Input Variables | Coefficient | P value | Coefficient | P value | Coefficient | P value | Coefficient | P value |
| Total INF - Australia (present) | 0.0054 | < 0.001 | 0.0050 | < 0.001 | 0.0036 | 0.001 | 0.0036 | < 0.001 |
| Total INF - Chile (present) | 0.0040 | 0.033 | 0.0020 | 0.251 | 0.0046 | 0.009 | 0.0021 | 0.196 |
| GT_INF A - Australia (present) | 0.0440 | 0.134 | 0.0524 | 0.062 | 0.0670 | 0.009 | 0.0762 | < 0.001 |
| GT_INF A - Chile (present) | 0.0035 | 0.769 | -0.0076 | 0.503 | -0.0322 | 0.002 | -0.0138 | 0.113 |
| Temp - U.S. (present) | 0.0101 | 0.326 | 0.0208 | 0.029 | 0.0274 | 0.006 | 0.0197 | 0.045 |
| Total INF - Australia (before 1 week) | -0.0024 | 0.120 | -0.0020 | 0.146 | -0.0016 | 0.216 | -0.0005 | 0.641 |
| Total INF - Chile (before 1 week) | 0.0013 | 0.566 | 0.0019 | 0.382 | 0.0009 | 0.706 | -0.0006 | 0.773 |
| GT_INF A - Australia (before 1 week) | -0.0093 | 0.867 | -0.0298 | 0.580 | -0.0870 | 0.074 | -0.0733 | 0.011 |
| GT_INF A - Chile (before 1 week) | 0.0150 | 0.505 | 0.0203 | 0.346 | 0.0314 | 0.118 | 0.0185 | 0.286 |
| Temp - U.S. (before 1 week) | 0.0085 | 0.534 | 0.0078 | 0.531 | 0.0149 | 0.250 | 0.0160 | 0.212 |
| Total INF - Australia (before 2 week) | -0.0039 | 0.011 | -0.0034 | 0.015 | -0.0022 | 0.089 | -0.0021 | 0.055 |
| Total INF - Chile (before 2 week) | -0.0008 | 0.737 | 0.0002 | 0.933 | 0.0017 | 0.453 | 0.0019 | 0.379 |
| GT_INF A - Australia (before 2 week) | -0.0542 | 0.333 | -0.0247 | 0.652 | 0.0432 | 0.379 | 0.0209 | 0.472 |
| GT_INF A - Chile (before 2 week) | -0.0579 | 0.013 | -0.0508 | 0.021 | -0.0294 | 0.159 | -0.0279 | 0.115 |
| Temp - U.S. (before 2 week) | -0.0006 | 0.963 | 0.0050 | 0.686 | 0.0103 | 0.428 | 0.0115 | 0.375 |
| Total INF - Australia (before 3 week) | 0.0045 | < 0.001 | 0.0036 | 0.001 | 0.0016 | 0.105 | 0.0001 | 0.943 |
| Total INF - Chile (before 3 week) | -0.0003 | 0.873 | -0.0012 | 0.408 | -0.0008 | 0.619 | 0.0011 | 0.450 |
| GT_INF A - Australia (before 3 week) | 0.0384 | 0.205 | 0.0134 | 0.648 | -0.0158 | 0.559 | 0.0031 | 0.857 |
| GT_INF A - Chile (before 3 week) | 0.0746 | < 0.001 | 0.0674 | < 0.001 | 0.0304 | 0.010 | 0.0235 | 0.022 |
| Temp - U.S. (before 3 week) | 0.0002 | 0.987 | -0.0034 | 0.721 | 0.0015 | 0.876 | 0.0108 | 0.269 |
| Total INF for the U.S. after 26 week – Output Variable | | | | | | | | |
| Adj.R-squared | 0.830 | | 0.842 | | 0.893 | | 0.917 | |
| Input Variables | Coefficient | P value | Coefficient | P value | Coefficient | P value | Coefficient | P value |
| Total INF - Australia (present) | -1.7 | 0.634 | -3.5 | 0.261 | -1.0 | 0.714 | 3.5 | 0.195 |
| Total INF - Chile (present) | 13.1 | 0.017 | 13.9 | 0.005 | 14.9 | 0.001 | -0.1 | 0.987 |
| GT_INF A - Australia (present) | 430.5 | < 0.001 | 271.0 | 0.001 | 281.9 | < 0.001 | 357.6 | < 0.001 |
| GT_INF A - Chile (present) | -20.5 | 0.542 | -47.8 | 0.131 | -57.4 | 0.023 | 0.1 | 0.998 |
| Total INF - Australia (before 1 week) | -5.3 | 0.230 | -6.0 | 0.128 | -5.3 | 0.106 | -3.6 | 0.293 |
| Total INF - Chile (before 1 week) | 6.3 | 0.361 | 10.1 | 0.105 | 7.0 | 0.221 | 3.7 | 0.589 |
| GT_INF A - Australia (before 1 week) | -289.3 | 0.076 | -141.8 | 0.363 | -302.6 | 0.015 | -274.5 | 0.003 |
| GT_INF A - Chile (before 1 week) | 19.5 | 0.767 | 37.4 | 0.547 | 17.8 | 0.728 | 11.2 | 0.835 |
| Total INF - Australia (before 2 week) | -2.9 | 0.516 | -3.2 | 0.422 | -2.3 | 0.482 | -5.7 | 0.095 |
| Total INF - Chile (before 2 week) | -3.5 | 0.598 | 4.6 | 0.446 | 5.1 | 0.364 | 6.1 | 0.371 |
| GT_INF A - Australia (before 2 week) | 16.5 | 0.920 | 51.9 | 0.743 | 146.6 | 0.242 | 95.2 | 0.298 |
| GT_INF A - Chile (before 2 week) | -91.5 | 0.177 | -106.5 | 0.094 | -41.2 | 0.440 | -77.6 | 0.162 |
| Total INF - Australia (before 3 week) | 8.8 | 0.008 | 8.4 | 0.006 | 5.3 | 0.031 | 2.2 | 0.327 |
| Total INF - Chile (before 3 week) | -6.8 | 0.149 | -8.7 | 0.041 | -5.6 | 0.160 | 9.5 | 0.033 |
| GT_INF A - Australia (before 3 week) | 22.5 | 0.797 | 11.5 | 0.891 | 42.4 | 0.537 | 58.7 | 0.272 |
| GT_INF A - Chile (before 3 week) | 165.3 | < 0.001 | 171.3 | < 0.001 | 124.1 | < 0.001 | 93.6 | 0.004 |
| INF A for the U.S. after 26 week – Output Variable | | | | | | | | |
| Adj.R-squared | 0.811 | | 0.825 | | 0.870 | | 0.903 | |
| Input Variables | Coefficient | P value | Coefficient | P value | Coefficient | P value | Coefficient | P value |
| INF A - Australia (present) | -2.5 | 0.553 | -1.8 | 0.620 | 2.1 | 0.451 | 5.3 | 0.048 |
| INF A - Chile (present) | 14.1 | 0.016 | 12.9 | 0.010 | 12.0 | 0.007 | -3.9 | 0.392 |
| GT_INF A - Australia (present) | 387.3 | < 0.001 | 249.2 | 0.001 | 246.6 | < 0.001 | 276.5 | < 0.001 |
| GT_INF A - Chile (present) | -19.1 | 0.562 | -48.2 | 0.101 | -46.3 | 0.046 | -4.5 | 0.837 |
| INF A - Australia (before 1 week) | -6.4 | 0.202 | -5.6 | 0.204 | -4.0 | 0.236 | -4.5 | 0.172 |
| INF A - Chile (before 1 week) | 5.5 | 0.465 | 6.8 | 0.292 | 4.6 | 0.432 | 2.5 | 0.698 |
| GT_INF A - Australia (before 1 week) | -256.5 | 0.110 | -134.3 | 0.355 | -243.0 | 0.034 | -205.9 | 0.005 |
| GT_INF A - Chile (before 1 week) | 14.2 | 0.826 | 42.2 | 0.462 | 29.6 | 0.529 | 22.6 | 0.614 |
| INF A - Australia (before 2 week) | -2.4 | 0.625 | -4.2 | 0.354 | -3.4 | 0.310 | -4.4 | 0.178 |
| INF A - Chile (before 2 week) | -4.0 | 0.581 | 3.9 | 0.539 | 4.5 | 0.436 | 7.9 | 0.211 |
| GT_INF A - Australia (before 2 week) | 78.4 | 0.627 | 69.1 | 0.640 | 136.2 | 0.240 | 57.7 | 0.435 |
| GT_INF A - Chile (before 2 week) | -85.0 | 0.202 | -103.3 | 0.078 | -44.6 | 0.364 | -62.5 | 0.175 |
| INF A - Australia (before 3 week) | 6.4 | 0.098 | 4.7 | 0.179 | -0.4 | 0.877 | -1.2 | 0.581 |
| INF A - Chile (before 3 week) | -2.1 | 0.676 | -3.7 | 0.391 | -0.2 | 0.968 | 9.3 | 0.023 |
| GT_INF A - Australia (before 3 week) | -48.8 | 0.581 | -26.4 | 0.741 | -4.0 | 0.950 | 28.7 | 0.503 |
| GT_INF A - Chile (before 3 week) | 150.1 | < 0.001 | 156.4 | < 0.001 | 100.3 | < 0.001 | 84.9 | 0.002 |
| INF B for the U.S. after 26 week – Output Variable | | | | | | | | |
| Adj.R-squared | 0.756 | | 0.712 | | 0.776 | | 0.851 | |
| Input Variables | Coefficient | P value | Coefficient | P value | Coefficient | P value | Coefficient | P value |
| INF B - Australia (present) | 4.1 | 0.019 | -1.6 | 0.410 | -3.4 | 0.153 | -1.6 | 0.555 |
| GT_INF A - Australia (present) | 43.4 | 0.005 | 44.2 | 0.044 | 74.7 | 0.001 | 79.6 | < 0.001 |
| INF B - Australia (before 1 week) | -1.4 | 0.530 | -3.0 | 0.218 | -3.1 | 0.297 | 1.2 | 0.716 |
| GT_INF A - Australia (before 1 week) | -33.6 | 0.274 | -35.2 | 0.418 | -83.8 | 0.063 | -62.8 | 0.074 |
| INF B - Australia (before 2 week) | -1.8 | 0.416 | 1.0 | 0.676 | 1.5 | 0.614 | -2.5 | 0.472 |
| GT_INF A - Australia (before 2 week) | -81.3 | 0.008 | -75.4 | 0.083 | -93.6 | 0.038 | 3.5 | 0.920 |
| INF B - Australia (before 3 week) | 3.9 | 0.024 | 11.3 | < 0.001 | 11.2 | < 0.001 | 5.2 | 0.032 |
| GT_INF A - Australia (before 3 week) | 113.3 | < 0.001 | 110.2 | < 0.001 | 174.8 | < 0.001 | 60.4 | 0.001 |

INF, Influenza; ILI, Influenza-like illness; GT, Google Trends; Temp, Temperature; U.S., United States of America
