# Supplementary material for: Forecasting type-specific seasonal influenza after 26 weeks in the United States using influenza activities in other countries
Source: PLoS One. 2019 Nov 25;14(11):e0220423. doi: 10.1371/journal.pone.0220423 (PMC6876883; doi:10.1371/journal.pone.0220423)
Supplement: S2 Table — (DOCX) [file pone.0220423.s002.docx]

**Supplementary Table 2.** Auto regressive integrated moving average models for influenza surveillance after 26 weeks in the U.S.

| ILI for the U.S. after 26 week – Output Variable | | | | | | | | |
| --- | --- | --- | --- | --- | --- | --- | --- | --- |
|  | Model for 2016 using 2010-2015 | | Model for 2017 using 2010-2016 | | Model for 2018 using 2010-2017 | | Model for 2019 using 2010-2018 | |
| AIC | 37.3 | | 15.4 | | 24.4 | | 41.8 | |
| ARIMAX (2,1,2) | Est. | P value | Est. | P value | Est. | P value | Est. | P value |
| AR(1) | 0.4041 | 0.745 | 0.504 | 0.789 | 0.503 | 0.999 | 0.550 | 0.111 |
| AR(2) | 0.1298 | 0.837 | 0.175 | 0.868 | 0.251 | 0.999 | 0.043 | 0.863 |
| MA(1) | 0.3283 | 0.788 | 0.306 | 0.870 | 0.358 | 0.999 | 0.486 | 0.151 |
| MA(2) | -0.1015 | 0.761 | -0.149 | 0.802 | -0.125 | 0.076 | 0.132 | 0.278 |
| Total INF - Australia (present) | 0.0036 | 0.001 | 0.0027 | 0.002 | 0.0016 | 0.019 | 0.001 | 0.115 |
| Total INF - Chile (present) | 0.0019 | 0.219 | 0.0013 | 0.306 | 0.0012 | 0.316 | -0.001 | 0.201 |
| GT_INF A - Australia (present) | -0.0197 | 0.422 | -0.0277 | 0.270 | -0.0110 | 0.503 | 0.026 | 0.004 |
| GT_INF A - Chile (present) | 0.0176 | 0.060 | 0.0117 | 0.173 | -0.0043 | 0.516 | 0.001 | 0.888 |
| Temp - U.S. (present) | 0.0069 | 0.386 | 0.0129 | 0.059 | 0.0121 | 0.056 | 0.013 | 0.026 |
| Total INF - Australia (before 1 week) | -0.0001 | 0.958 | 0.0000 | 0.994 | -0.0005 | 0.492 | 0.000 | 0.941 |
| Total INF - Chile (before 1 week) | 0.0024 | 0.126 | 0.0024 | 0.183 | 0.0017 | 0.126 | 0.001 | 0.508 |
| GT_INF A - Australia (before 1 week) | 0.0522 | 0.102 | 0.0605 | 0.035 | -0.0016 | 0.940 | -0.004 | 0.739 |
| GT_INF A - Chile (before 1 week) | -0.0071 | 0.606 | -0.0060 | 0.724 | 0.0068 | 0.436 | 0.003 | 0.666 |
| Temp - U.S. (before 1 week) | 0.0096 | 0.226 | 0.0120 | 0.073 | 0.0156 | 0.012 | 0.019 | 0.001 |
| Total INF - Australia (before 2 week) | -0.0022 | 0.029 | -0.0020 | 0.013 | -0.0015 | 0.023 | -0.002 | 0.005 |
| Total INF - Chile (before 2 week) | 0.0007 | 0.660 | 0.0013 | 0.273 | 0.0020 | 0.066 | 0.004 | < 0.001 |
| GT_INF A - Australia (before 2 week) | -0.0272 | 0.388 | -0.0112 | 0.745 | 0.0238 | 0.237 | 0.009 | 0.425 |
| GT_INF A - Chile (before 2 week) | -0.0251 | 0.074 | -0.0201 | 0.126 | -0.0092 | 0.299 | -0.004 | 0.530 |
| Temp - U.S. (before 2 week) | 0.0034 | 0.667 | 0.0071 | 0.298 | 0.0129 | 0.041 | 0.018 | 0.001 |
| Total INF - Australia (before 3 week) | 0.0014 | 0.183 | 0.0009 | 0.429 | 0.0000 | 0.952 | -0.001 | 0.084 |
| Total INF - Chile (before 3 week) | 0.0006 | 0.670 | 0.0005 | 0.740 | 0.0012 | 0.236 | 0.002 | 0.057 |
| GT_INF A - Australia (before 3 week) | 0.0154 | 0.514 | -0.0059 | 0.811 | 0.0061 | 0.726 | 0.015 | 0.159 |
| GT_INF A - Chile (before 3 week) | 0.0481 | < 0.001 | 0.0394 | 0.001 | 0.0198 | 0.011 | 0.007 | 0.278 |
| Temp - U.S. (before 3 week) | -0.0008 | 0.920 | -0.0008 | 0.907 | 0.0054 | 0.397 | 0.007 | 0.229 |
| Total INF for the U.S. after 26 week – Output Variable | | | | | | | | |
| AIC | 3373.6 | | 4195.8 | | 5068.4 | | 6035.5 | |
| ARIMAX (2,1,2) | Est. | P value | Est. | P value | Est. | P value | Est. | P value |
| AR(1) | 0.802 | < 0.001 | -0.144 | < 0.001 | 0.859 | < 0.001 | 1.058 | < 0.001 |
| AR(2) | -0.031 | 0.866 | 0.846 | < 0.001 | -0.198 | 0.071 | -0.442 | < 0.001 |
| MA(1) | 0.869 | < 0.001 | 1.573 | < 0.001 | 0.438 | < 0.001 | 0.263 | < 0.001 |
| MA(2) | 0.516 | < 0.001 | 0.573 | < 0.001 | 0.391 | < 0.001 | 0.515 | < 0.001 |
| Total INF - Australia (present) | -1.5 | 0.225 | -2.8 | 0.022 | -2.5 | 0.036 | -0.1 | 0.956 |
| Total INF - Chile (present) | 6.2 | 0.006 | 11.2 | < 0.001 | 4.0 | 0.068 | 0.0 | 0.997 |
| GT_INF A - Australia (present) | -29.4 | 0.366 | -7.2 | 0.821 | 35.7 | 0.255 | 92.9 | < 0.001 |
| GT_INF A - Chile (present) | 23.0 | 0.094 | 10.0 | 0.423 | -25.3 | 0.037 | -4.3 | 0.735 |
| Total INF - Australia (before 1 week) | -1.1 | 0.445 | -3.4 | 0.017 | -1.6 | 0.185 | -1.3 | 0.256 |
| Total INF - Chile (before 1 week) | 7.2 | 0.005 | 9.4 | < 0.001 | 4.2 | 0.046 | 6.6 | 0.001 |
| GT_INF A - Australia (before 1 week) | 106.2 | < 0.001 | 102.2 | 0.003 | -15.4 | 0.625 | 22.9 | 0.274 |
| GT_INF A - Chile (before 1 week) | 2.5 | 0.857 | -10.4 | 0.436 | 19.3 | 0.162 | 38.3 | 0.003 |
| Total INF - Australia (before 2 week) | -1.7 | 0.235 | -2.5 | 0.071 | -0.3 | 0.820 | -1.1 | 0.289 |
| Total INF - Chile (before 2 week) | 5.0 | 0.020 | 9.6 | < 0.001 | 9.1 | < 0.001 | 15.4 | < 0.001 |
| GT_INF A - Australia (before 2 week) | 89.8 | 0.003 | 53.6 | 0.110 | 102.1 | 0.001 | 86.4 | < 0.001 |
| GT_INF A - Chile (before 2 week) | 9.3 | 0.494 | -12.3 | 0.365 | 24.7 | 0.089 | -13.8 | 0.330 |
| Total INF - Australia (before 3 week) | -0.4 | 0.750 | 2.2 | 0.055 | 0.3 | 0.765 | -3.5 | < 0.001 |
| Total INF - Chile (before 3 week) | 4.3 | 0.018 | 3.4 | 0.046 | 4.7 | 0.014 | 10.1 | < 0.001 |
| GT_INF A - Australia (before 3 week) | -9.6 | 0.758 | 14.2 | 0.665 | 33.1 | 0.282 | 38.7 | 0.102 |
| GT_INF A - Chile (before 3 week) | 16.7 | 0.251 | 39.8 | 0.003 | 34.4 | 0.017 | -22.3 | 0.145 |
| INF A for the U.S. after 26 week – Output Variable | | | | | | | | |
| AIC | 3340.5 | | 4134.8 | | 4975.4 | | 5891.1 | |
| ARIMAX (2,1,2) | Est. | P value | Est. | P value | Est. | P value | Est. | P value |
| AR(1) | 0.588 | < 0.001 | 1.510 | < 0.001 | 0.926 | < 0.001 | 1.036 | < 0.001 |
| AR(2) | 0.160 | 0.168 | -0.659 | < 0.001 | -0.236 | 0.023 | -0.388 | < 0.001 |
| MA(1) | 1.147 | < 0.001 | 0.039 | 0.811 | 0.437 | < 0.001 | 0.297 | < 0.001 |
| MA(2) | 0.744 | < 0.001 | -0.006 | 0.960 | 0.408 | < 0.001 | 0.515 | < 0.001 |
| INF A - Australia (present) | -2.4 | 0.094 | -3.1 | 0.021 | -1.4 | 0.233 | 0.4 | 0.709 |
| INF A - Chile (present) | 6.8 | 0.001 | 5.6 | 0.006 | 0.1 | 0.976 | -2.8 | 0.150 |
| GT_INF A - Australia (present) | -50.0 | 0.090 | -6.1 | 0.835 | 32.6 | 0.248 | 68.9 | < 0.001 |
| GT_INF A - Chile (present) | 16.9 | 0.137 | 35.4 | 0.005 | -10.2 | 0.342 | 0.6 | 0.958 |
| INF A - Australia (before 1 week) | -2.5 | 0.164 | -3.4 | 0.037 | -2.3 | 0.072 | -2.0 | 0.078 |
| INF A - Chile (before 1 week) | 7.6 | 0.001 | 4.2 | 0.062 | 3.0 | 0.140 | 5.4 | 0.003 |
| GT_INF A - Australia (before 1 week) | 112.2 | < 0.001 | 135.5 | < 0.001 | 16.7 | 0.536 | 17.9 | 0.267 |
| GT_INF A - Chile (before 1 week) | -8.0 | 0.470 | 11.7 | 0.337 | 25.2 | 0.028 | 36.7 | 0.001 |
| INF A - Australia (before 2 week) | -2.2 | 0.213 | -2.8 | 0.077 | -2.5 | 0.040 | -1.8 | 0.067 |
| INF A - Chile (before 2 week) | 4.2 | 0.049 | 8.4 | < 0.001 | 8.8 | < 0.001 | 13.7 | < 0.001 |
| GT_INF A - Australia (before 2 week) | 98.4 | < 0.001 | 46.2 | 0.116 | 93.3 | < 0.001 | 74.0 | < 0.001 |
| GT_INF A - Chile (before 2 week) | 19.6 | 0.088 | -18.7 | 0.146 | 16.2 | 0.184 | -8.9 | 0.451 |
| INF A - Australia (before 3 week) | -1.0 | 0.424 | 1.3 | 0.327 | -1.8 | 0.103 | -3.5 | 0.000 |
| INF A - Chile (before 3 week) | 5.3 | 0.003 | 5.6 | 0.001 | 7.2 | < 0.001 | 10.8 | < 0.001 |
| GT_INF A - Australia (before 3 week) | -18.1 | 0.526 | 7.0 | 0.816 | 4.6 | 0.869 | 3.8 | 0.846 |
| GT_INF A - Chile (before 3 week) | 20.1 | 0.110 | 5.9 | 0.656 | 24.4 | 0.049 | -10.3 | 0.415 |
| INF B for the U.S. after 26 week – Output Variable | | | | | | | | |
| AIC | 2479.5 | | 3218.3 | | 4130.5 | | 5026.6 | |
| ARIMAX (1,1,2) | Est. | P value | Est. | P value | Est. | P value | Est. | P value |
| AR(1) | 0.947 | < 0.001 | 0.938 | < 0.001 | 0.899 | < 0.001 | 0.801 | < 0.001 |
| MA(1) | 0.552 | < 0.001 | 0.615 | < 0.001 | 0.586 | < 0.001 | 0.667 | < 0.001 |
| MA(2) | 0.271 | < 0.001 | 0.180 | < 0.001 | 0.388 | < 0.001 | 0.542 | < 0.001 |
| INF B - Australia (present) | 0.756 | 0.107 | -0.6 | 0.205 | -0.4 | 0.608 | 0.705 | 0.404 |
| GT_INF A - Australia (present) | -2.570 | 0.533 | -10.1 | 0.059 | -3.9 | 0.571 | 21.162 | < 0.001 |
| INF B - Australia (before 1 week) | 1.392 | 0.012 | 0.5 | 0.376 | 1.7 | 0.064 | 2.879 | 0.002 |
| GT_INF A - Australia (before 1 week) | -1.219 | 0.768 | 8.1 | 0.136 | -17.2 | 0.018 | -2.261 | 0.690 |
| INF B - Australia (before 2 week) | 0.502 | 0.355 | 1.9 | 0.002 | 4.1 | < 0.001 | 2.981 | 0.001 |
| GT_INF A - Australia (before 2 week) | 7.069 | 0.098 | 8.7 | 0.109 | 18.4 | 0.015 | 12.869 | 0.021 |
| INF B - Australia (before 3 week) | 0.375 | 0.417 | 2.6 | < 0.001 | 3.9 | < 0.001 | 1.083 | 0.138 |
| GT_INF A - Australia (before 3 week) | 6.277 | 0.137 | 10.1 | 0.062 | 23.6 | 0.002 | 26.531 | < 0.001 |

ARIMAX, Auto Regressive Integrated Moving Average including exogenous variables; AIC, Akaike’s Information Criterion; AR, Autoregressive Coefficients; MA, Moving Average Coefficients; Est., Estimated values through conditional least square method; INF, Influenza; ILI, Influenza-like illness; GT, Google Trends; Temp, Temperature; U.S., United States of America
